# Pancreatic pseudocyst or mucinous cystadenocarcinoma of pancreas? A diagnostic dilemma

**DOI:** 10.1002/ccr3.887

**Published:** 2017-03-06

**Authors:** Utsav Joshi, Prakash Poudel, Ram Kumar Ghimire, Babin Basnet

**Affiliations:** ^1^ Maharajgunj Medical CampusInstitute of MedicineKathmanduNepal; ^2^ Department of RadiologyInstitute of MedicineKathmanduNepal

**Keywords:** Cystic neoplasms, mucinous cystadenocarcinoma, pancreas, pseudocyst

## Abstract

Pancreatic cystic neoplasm is difficult to distinguish from pseudocyst as clinical and radiological evidences may not be sufficient to make an accurate diagnosis. This may result in misdiagnosis with inappropriate management. Hence, every effort should be made for their distinction to avoid internal drainage procedures for neoplasms instead of extirpation.

## Introduction

Cystic lesions of the pancreas comprise a heterogeneous group of diagnostic entities, which may be either benign or malignant. Pseudocysts of the pancreas are the commonest form of cystic lesions that often develop in patients of chronic pancreatitis and less commonly, in patients of acute pancreatitis. However, neoplastic cysts are not as common as pseudocysts and only 15% of the pancreatic cysts are neoplastic, benign, or malignant 1. Cystic neoplasms, being quite rare, are frequently misdiagnosed as pseudocysts. On the one hand, pseudocysts are managed with drainage procedures, either internally into the gastrointestinal tract or externally and excision is usually not possible. On the other hand, cystic neoplasms of the pancreas must be excised as appropriate and drainage procedure for the neoplastic cysts is inappropriate and devastating. Hence, differentiation between the two conditions is of utmost importance for appropriate management.

We present a case of mucinous cystadenocarcinoma of the pancreas which was initially misdiagnosed as pancreatic pseudocyst but the correct diagnosis was made later by which time, the disease had already metastasized.

## Case Report

A sixty‐five‐year‐old female patient was admitted to the department of surgery in a university hospital with the complaint of an abdominal mass in the left hypochondrium for 2 months. The abdominal mass was initially localized in the left hypochondrium but gradually increased in size to progress toward the epigastric region. The mass was associated with abdominal pain and multiple episodes of nonbilious, non‐blood‐mixed vomiting. There was no history of fever, jaundice, melena, or hematemesis. She did not have any history of weight loss or loss of appetite.

The patient had a prior history of multiple episodes of abdominal pain accompanied by vomiting 3 months earlier for which she was managed conservatively with analgesics, antibiotics, and antiemetics. Following third episode of abdominal pain, she felt the presence of a lump in her left upper quadrant.

She was a nonsmoker but consumed nearly 500 mL of homemade alcohol (nearly 15% volume/volume) every day for the last 40 years.

Clinical examination revealed slightly distended abdomen with a mass felt in the epigastric region measuring 15 × 10 cm which was soft, nontender, and not moving with respiration. There was no shifting dullness or fluid thrill and bowel sounds were heard all over the abdomen.

Hematological investigations, blood sugar level, renal function tests, liver function tests, serology, blood and urine culture, amylase and lipase levels were within normal limits.

Ultrasonography of the abdomen was performed which showed large well‐defined cystic appearing mass lesion measuring approximately 15 × 12.7 × 11.3 cm (volume = 1139.97 cc) in the left upper quadrant, retroperitoneal in location and that abutted the body and tail of pancreas supero‐medially. A provisional diagnosis of pancreatic pseudocyst was made.

**Figure 1:**
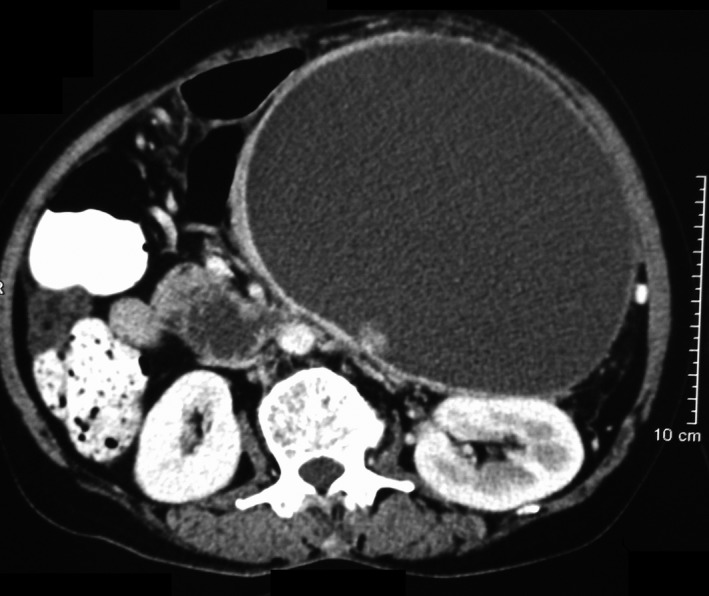
CECT axial image shows a heterogeneously enhancing mural nodule in a large cyst of pancreas arising from the body and tail.

**Figure 2:**
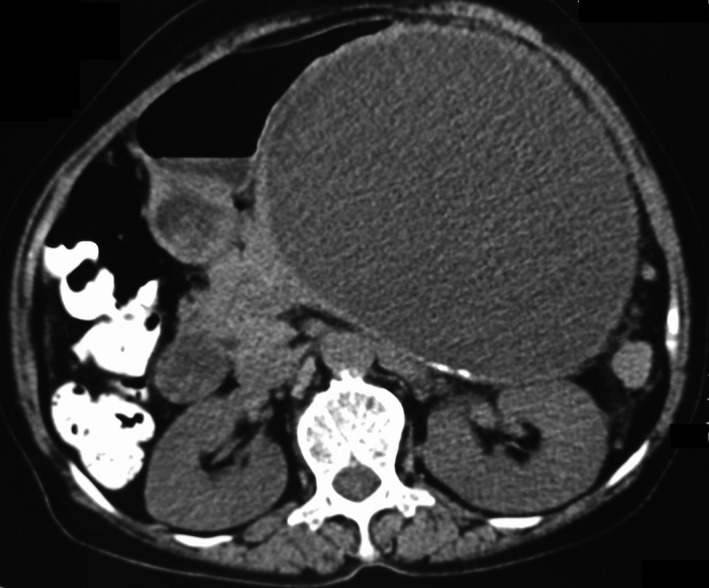
Noncontrast axial image shows wall calcification of the pancreatic cyst.

**Figure 3:**
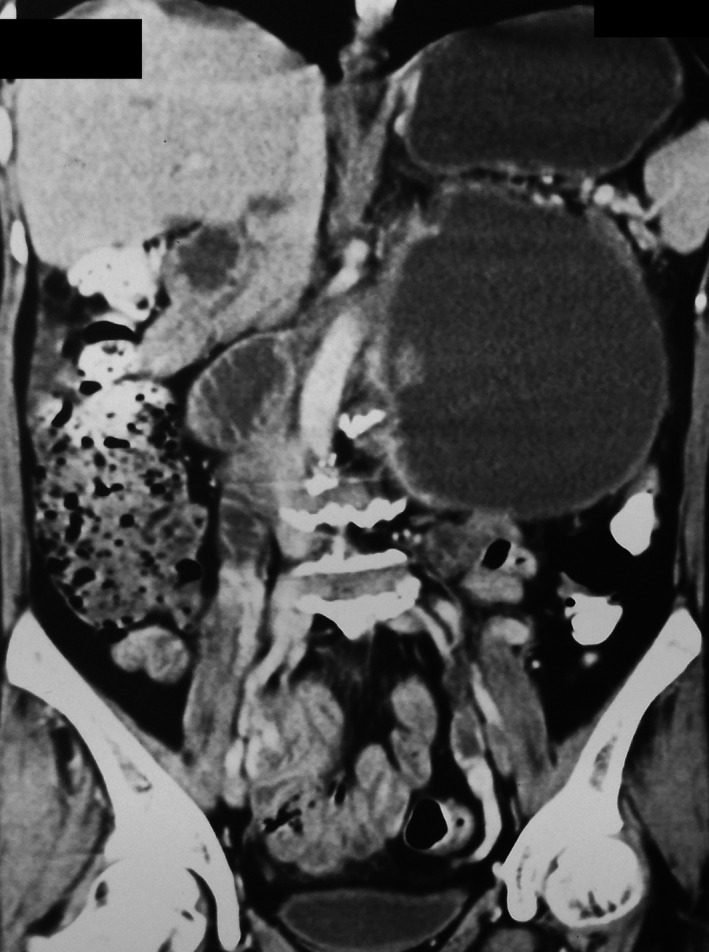
CECT coronal image shows peripherally enhancing pancreatic cyst with heterogeneously enhancing mural nodule.

**Figure 4:**
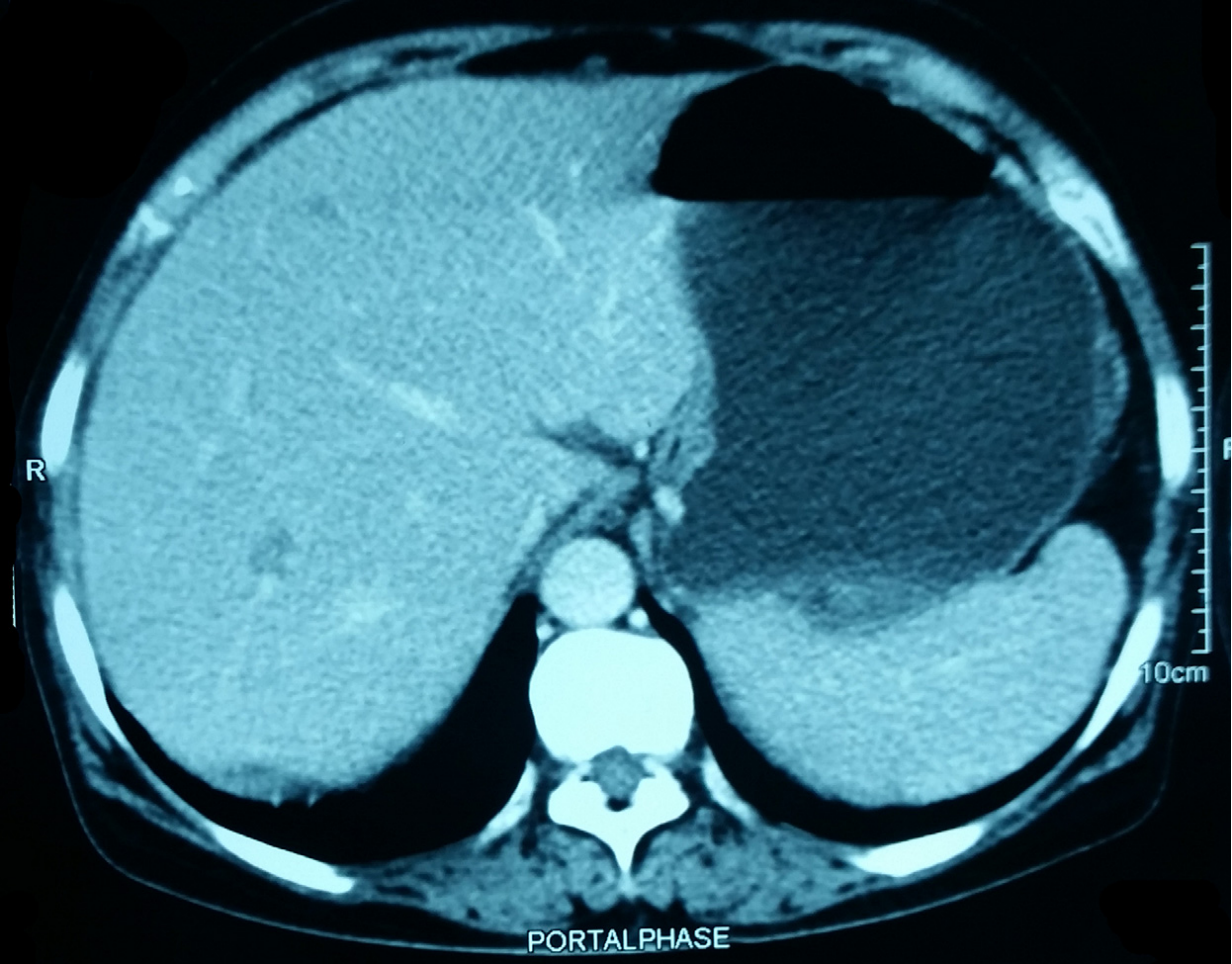
Peripherally enhancing, irregular, marginated, subcentimeter lesion in segment IVa, and segment VII of liver‐ suspicious of metastasis.

A contrast‐enhanced computed tomography (CECT) of the abdomen (Figs 1, 2, 3, 4) was planned for further confirmation of pancreatic pseudocyst. The CECT scan showed a large, well‐defined, enhancing thin‐walled unilocular cystic lesion 14 × 14 × 16 cm in size arising from body and tail of pancreas. Enhancing solid nodule and peripheral areas of calcification with an indeterminate nodule in the liver was also observed, which was feature suggestive of mucinous cystic neoplasm of pancreas. There was no communication of the cystic lesion with main pancreatic duct.

Her blood CEA and CA 19‐9 levels were 5.2 ng/mL and 873 U/mL, respectively, both of which were above the normal range.

A final diagnosis of mucinous cystic neoplasm of pancreas was made, and the patient was planned for exploratory laparotomy. Operative finding was a huge cyst arising from the body and tail of the pancreas. The cyst was well defined and pushed the stomach anteriorly. Multiple metastases were present in the liver, omentum, and bowel, and hence, only biopsy was taken from cystic lesion and omentum. Histopathological examination revealed mucinous cystadenocarcinoma of the pancreas with peritoneal metastasis.

## Discussion

This case report depicts similarities in presentations of both pancreatic pseudocyst and mucinous cystadenocarcinoma of the pancreas. Because of these similarities, often wrong diagnosis is made which results in faulty intervention with devastating complications.

Cystic tumors of the pancreas account for 15% of all pancreatic cysts but only 1% of all pancreatic malignancies 1, 2. This malignant tumor almost exclusively affects middle‐aged women especially at an age of <50 years and predominantly appears in the body or tail of the pancreas, although occasionally, may occur in the head 2, 3.

Pancreatic pseudocysts comprise 75% of cystic lesions of pancreas 4; 5–10% of patients with acute pancreatitis and up to 50% of patients with chronic pancreatitis may develop pancreatic pseudocyst 4. Pseudocyst may occur in any gender as opposed to the female preponderance of the mucinous cystadenocarcinoma and it does not have any favorable location in the pancreas.

The presentations of mucinous cystadenocarcinoma are similar to that of pancreatic pseudocyst. Patients may present with epigastric pain, postprandial fullness, palpable abdominal mass, nausea, vomiting, diarrhea, steatorrhea, and/or weight loss 2. Majority of the cases of pancreatic pseudocyst present with prior history of acute pancreatitis. With a history of acute pancreatitis, surgeons often tend to diagnose newly discovered pancreatic cyst as a pseudocyst. Nevertheless, the possibility of a cystic neoplasm should always be taken into account 4. In this case, prior history of abdominal pain suggested a diagnosis of pancreatic pseudocyst in the beginning, but, later, evidences pointed toward mucinous cystic neoplasm.

Imaging with computed tomography scan (CT) and magnetic resonance imaging (MRI) can be useful to differentiate pancreatic cystic neoplasm from pseudocyst. Presence of multiple cysts and internal septations are highly suggestive of tumor 2, 5. Besides imaging, CEA is a highly sensitive tumor marker to differentiate between mucinous and nonmucinous cyst 2. Cyst fluid amylase level is elevated in approximately 50–75% of the patients of pancreatic pseudocysts, with pseudocyst being effectively ruled out if amylase level is below 250 U/L 5. Biopsy of the cyst wall with frozen section examination should always be performed to differentiate pseudocyst from cystic neoplasms 4, 5.

In a large series of patients with resectable mucinous cystadenocarcinoma of the pancreas, a 5‐year survival of 63% was reported 6. In another similar study, 62% of the patients had no disease recurrence after a mean follow‐up of 61 months 7. Hence, survival following surgical resection was better as compared to nonresected patients with mucinous cystadenocarcinoma 8.

For pancreatic pseudocysts, surgery can be planned after 4–6 weeks of symptom onset. During this period, maturation of fibrous wall occurs that allows easier internal drainage 9. Conservative management of pseudocysts has a low success rate (between 3% and 39%) when they are symptomatic and greater than 6 cm in size 10. Hence, pseudocysts that are asymptomatic and <6 cm in size can simply be followed by CT at 3‐ to 6‐month intervals. In rest of the cases, therapeutic interventions like cystoenterostomy should be considered 11.

In the presented case, the patient had multiple episodes of abdominal pain for many months prior to her presentation. This chronic history of abdominal pain was followed by the development of an abdominal mass. Besides, the patient was a chronic alcohol consumer. These clinical evidences combined with the ultrasonography findings supported the diagnosis of pancreatic pseudocyst that most probably developed on the background of chronic pancreatitis. CECT, however, showed enhancing solid nodule and areas of calcifications within the cystic lesion that suggested mucinous cystic neoplasm. This was further supported by the elevated level of CEA and CA 19‐9 in our patient. On exploration, the operating surgeons found evidences of metastases in liver, omentum and bowel and hence, decided to take the biopsy samples without radical resection of the tumor. In situations like this, the failure to correctly identify the cystic lesion as either pseudocyst or cystic neoplasm might result in serious complications. In case neoplastic lesions are incorrectly diagnosed as pancreatic pseudocysts and internal drainage procedures performed, this may result in inadequate control of symptoms, development of painful ulcers in the gastrointestinal tract with potential chances of infection, and also fail to cure a curable cancer with potential of future metastasis.

## Conclusion

Our case report highlights the fact that pancreatic pseudocyst and cystic neoplasm of the pancreas can be very difficult to distinguish from one another. Clinical evidences and radiological evaluations may not be specific enough to make a diagnosis. In our case, recurrent episodes of abdominal pain followed by the development of an abdominal mass led us to believe it as a case of pancreatic pseudocyst. But further investigations were not consistent with pancreatic pseudocyst. Hence, every effort should be made to make a correct diagnosis because of the differences in their management modalities.

## Conflict of Interest

The authors declare that they have no competing interests.

## Authorship

UJ: Conception and design, Acquisition of data, Analysis and interpretation of data, drafting the article, and final approval of the version to be published. PP: Conception and design, Acquisition of data, Analysis and interpretation of data, drafting the article, and final approval of the version to be published. RKG: Conception and design, critical revision of the article, and final approval of the version to be published. BB: Acquisition of data, Analysis and interpretation of data, drafting the article, and final approval of the version to be published.
